# Letter to the editor regarding “Comparison of a twin interlocking derotation and compression screw cephalomedullary nail (InterTAN) with a single screw derotation cephalomedullary nail (proximal femoral nail antirotation): a systematic review and meta-analysis for intertrochanteric fractures”

**DOI:** 10.1186/s13018-022-03174-6

**Published:** 2022-05-21

**Authors:** Hengda Hu, Yujian Hui

**Affiliations:** Department of Orthopedics, Jiangyin People’s Hospital, 3 Yingrui Road, Jiangyin, 214400 Jiangsu People’s Republic of China

Dear Editor

We recently read an article written by Leo Nherera et al. [1]. The authors compared the clinical outcomes of InterTAN with PFNA in intertrochanteric hip fractures. We acknowledge their contribution to this field, and they received some insightful conclusions; however, some controversies need to be clarified.

First, the research conducted by Yu et al. [2] was actually a meta-analysis, rather than a retrospective observational study as the authors claimed. Besides, no patients were treated with InterTAN in Yu's article. There must be a mistake that needs to be fixed.

Second, in the “Study selection and eligibility criteria” section, the inclusion criteria were described as “Adults with intertrochanteric hip fractures with subtrochanteric extension or subtrochanteric fractures,” which did not correspond to the purpose of this article, because subtrochanteric fractures are much different from intertrochanteric fractures, not to mention that this leads to a greater heterogeneity.

Third, only 188 patients were recruited in 2 RCTs and these cases were far from enough, given this meta-analysis involved 987 cases and 6 researches. Furthermore, although the authors claimed RCTs were assessed with the Cochrane Collaboration's risk of bias tool, the risk of bias graph was not found in this study. Meanwhile, the Newcastle–Ottawa Scale [3] was usually applied for observational research, the authors claimed they use a GRACE checklist instead, which is not common in most meta-analysis. Despite this, the result of the GRACE checklist was not attached in this article.

Fourth, the included researches were either conducted in Turkey or China; however, the authors did not mention ethnic bias which could be critical to the conclusion. Also, only 2 or 3 included researches were applied in some subgroup analysis.

The controversies above may weaken the reliability of this meta-analysis. As a result, more studies need to be carried out to clarify these issues.

## Data Availability

Not applicable.
